# Autism with co-occurring epilepsy care pathway in Europe

**DOI:** 10.1192/j.eurpsy.2023.2426

**Published:** 2023-07-20

**Authors:** Maria A. Mendez, Roberto Canitano, Bethany Oakley, Antonia San José-Cáceres, Michela Tinelli, Martin Knapp, James Cusack, Mara Parellada, Pierre Violland, Jan R. Derk Plas, Declan G. M. Murphy, Vinciane Quoidbach, Celso Arango

**Affiliations:** 1 Instituto de Investigación Sanitaria Gregorio Marañón, Hospital General Universitario Gregorio Marañón, Madrid, Spain; 2 Department of Psychiatry, Azienda ospedaliero-universitaria Senese, Siena, Italy; 3 Department of Forensic and Neurodevelopmental Sciences, Institute of Psychiatry, Psychology and Neuroscience, Kings College London, London, UK; 4 AIMS-2-TRIALS Consortium; 5 Mental Health Networking Biomedical Research Centre (CIBERSAM), Instituto Salud Carlos III, Madrid, Spain; 6 Department of Health Policy, London School of Economics and Political Science, London, UK; 7 Autistica, London, UK; 8 Department of Child and Adolescent Psychiatry, Institute of Psychiatry and Mental Health, Hospital General Universitario Gregorio Marañón, Madrid, Spain; 9 School of Medicine, Universidad Complutense, Madrid, Spain; 10 European Brain Council, Brussels, Belgium

**Keywords:** autism, co-occurring epilepsy, harmonized guidance, policy recommendations, screening/diagnosis, treatment

## Abstract

**Background:**

Autism and epilepsy often occur together. Epilepsy and other associated conditions have a substantial impact on the well-being of autistic people and their families, reduce quality of life, and increase premature mortality. Despite this, there is a lack of studies investigating the care pathway of autistic children with co-occurring epilepsy in Europe.

**Methods:**

We analyzed the care pathway for autistic children with associated epilepsy in Italy, Spain, and the United Kingdom from the perspective of caregivers (using a survey aimed at caregivers of autistic children 0–18 years old), the autistic community, and professionals, in order to identify major barriers preventing caregivers and autistic children from receiving timely screening and treatment of possible co-occurring epilepsy.

**Results:**

Across all three countries, an analysis of the current care pathway showed a lack of systematic screening of epilepsy in all autistic children, lack of treatment of co-occurring epilepsy, and inappropriate use of antiepileptic drugs. A major challenge is the lack of evidence-based harmonized guidelines for autism with co-occurring epilepsy in these countries.

**Conclusions:**

Our findings show both heterogeneity and major gaps in the care pathway for autism with associated epilepsy and the great efforts that caregivers must make for timely screening, diagnosis, and adequate management of epilepsy in autistic children. We call for policy harmonization in Europe in order to improve the experiences and quality of life of autistic people and their families.

## Introduction

Some studies estimate the prevalence of autism in Europe to be between 1% and 2% in school-aged children, making it a common childhood condition [1, 2]. The prevalence of epilepsy in autism has been reported to be greater than in the general population, but estimated rates vary greatly depending on the population, study methods, concomitant predisposing factors, and clinical characteristics [3–5].

The co-occurrence of epilepsy in autism is well documented. In a meta-analysis evaluating the occurrence of epilepsy in both children and adults with autism, epilepsy was concomitant in 21.4% of people with autism who also had a diagnosis of intellectual disability (ID) and 8% in autistic individuals without an ID [6]. Similarly, another meta-analysis reported an epilepsy prevalence of 23.7% in autistic individuals over the age of 12 with an ID compared with 8.9% in autistic individuals without an ID and a prevalence of 1% in the general non-autistic population [7, 8]. A recent systematic review and meta-analysis evaluated the prevalence of epilepsy in autistic individuals and the putative factors influencing it. It reported a pooled prevalence of epilepsy of 7% in autistic children and 19% in autistic adults, and this rate increased with age. Prevalence of epilepsy was also significantly higher in females and autistic individuals with low intellectual function [8]. Additionally, in a study investigating the independent role of four autism severity measures (ID, language impairment, core autism symptoms severity, and motor dysfunction) and controlling for age and sex, each one of these severity factors independently predicted a small increased risk for epilepsy [9].

Studies point to the co-occurrence of autism and epilepsy as the result of common divergent neurodevelopmental pathways, and there is evidence that shared neurobiological mechanisms are responsible for the co-occurrence of epilepsy, autism, and ID. Furthermore, the mechanisms that lead to epilepsy may also interfere with social development and overall cognition [3]. For example, a study using the Social Responsiveness Scale [10] found that autistic individuals with epilepsy experienced more difficulties than autistic participants without epilepsy in measures of social functioning such as social awareness and social communication [11].

Electroencephalography (EEG) has been used to investigate epilepsy in autism [12]. EEG in autism is usually administered during the diagnostic stage and during follow-up. Even though epileptiform abnormalities with and without clinically obvious seizures have been described in as many as 20%–30% of autistic individuals [13], there is no consensus regarding the time and frequency EEG assessment should be conducted. Moreover, current clinical guidelines do not recommend routine electroencephalograms (EEGs) as a screening tool for epileptic activity in autistic children unless there is clinical suspicion [14–16]. However, the clinical identification of seizures in autism is challenging as its presentation can be complicated by the presence of repetitive body movements and behavioral features in autism [17, 18].

Co-occurring conditions such as epilepsy have a substantial impact on the well-being and outcomes of autistic people and their families across developmental stages, reducing quality of life and increasing premature mortality [19, 20]. Nevertheless, there is a current lack of studies assessing the care pathway and experiences of autistic people and co-occurring epilepsy and their families in Europe.

In 2016, the European Brain Council (EBC), an organization promoting research on brain health and disorders in Europe, initiated a pan-European study called the Value of Treatment (VoT) for Brain Disorders. In 2018, in a second round (VoT2), the EBC added case studies on neurodevelopmental conditions including autism. The VoT2 project aims to examine the value of early diagnosis and intervention, the benefits of coordinated care and multidisciplinary care on patient outcomes, and the social and economic impacts resulting from the best practice healthcare interventions in comparison with current care or no treatment, and to offer evidence to policymakers. In this paper, we present findings on the journey of caregivers of autistic children and adolescents and co-occurring epilepsy. Our objectives are (1) to identify the current treatment gaps and needs of autistic children with epilepsy and the underlying causes of these gaps and (2) to propose policy recommendations on how to improve this care pathway. We hope that our findings will raise policy awareness and inform policy harmonization in Europe, with the aim of improving the screening, diagnosis, and the effective early treatment of comorbid epilepsy, thus improving the lives of autistic people and their families.

## Methods

In order to identify major barriers and treatment gaps preventing parents or caregivers of autistic children from receiving timely screening, diagnosis, and treatment of possible co-occurring epilepsy, we analyzed the care pathway of autistic children and adolescents with co-occurring epilepsy. We carried out a rapid literature review on the existing care pathway in Europe for autistic people and co-occurring epilepsy and developed a survey aimed at parents or caregivers of autistic children aged 0–18 living in Italy, Spain, or the United Kingdom. Additionally, we met in Brussels and remotely (due to the COVID-19 pandemic) between 2019 and 2021 to identify the main treatment gaps and their causes, discuss the survey results, and propose policy recommendations.

### Survey development

We conducted an online survey based on the one developed by the Autism Spectrum Disorder in the European Union network [21] with some modifications. Our survey collected information about caregiver access and experiences with local services from the moment they noticed difficulties with their children’s behavior and/or neurodevelopment (first concerns), the time an autism diagnosis was confirmed, if an EEG was done, and the time of the EEG, if the autistic child was diagnosed with associated epilepsy and if epilepsy was treated. For further details about sample characteristics and specific survey questions regarding epilepsy please refer to Supplementary material.

### Inclusion criteria

The survey was aimed at parents, caregivers, or legal representatives of autistic children aged 0–18 years resident in Italy, Spain, and the United Kingdom. Only participants that read the information sheet and consented to participate in this study were able to complete the survey. The reasons for exclusion were: having an autistic child older than 18, not being a resident in one of the three countries or not signing the online informed consent.

### Recruitment procedure

Participants were identified from the researchers’ institutions, organizations for autistic people, and their families and professional organizations related to autism. The survey was made available online and disseminated through social networks using the link generated by the REDCap platform. An invitation and summary information about the study was included with this link.

### Ethical approval

Ethical approval was given by Ethics Committee of the Hospital General Universitario Gregorio Marañón, Spain (VoTASD 391/20), by King’s College London Research Ethics Management, UK (LRS-20/21-21196) and by the Ethics Committee of the Azienda ospedaliera-universitaria Senese, Italy.

### Data analysis

The survey was administered electronically, and the data were downloaded and transferred for further analysis. Comprehensive descriptive analyses were performed using Statistical Package for the Social Sciences version 25 [22]. Analyses consisted of the description of the sample using means, medians, standard deviations, and percentages in order to describe subgroups.

For further information about methodology and access to complete survey, please refer to the Autism care pathway in Europe manuscript by Mendez et al. included in this edition.

## Results

### Sample characteristics

A total of 712 people began the survey, and 663 (158 from Italy, 287 from Spain, and 218 from the United Kingdom) met the inclusion criteria. See Table 1.

**Table 1.** Sample characteristics

Sample characteristics	
Age of respondents in years, mean (SD)	44 (8.08)
Sex of person completing survey (% male, % female)	16%, 84%
Autistic child’s age at time of survey, mean (SD)	10 (4.39)
Sex of autistic child (% male, % female)	77%, 23%

### Specialist assistance during the autism diagnosis process

**Figure 1.**
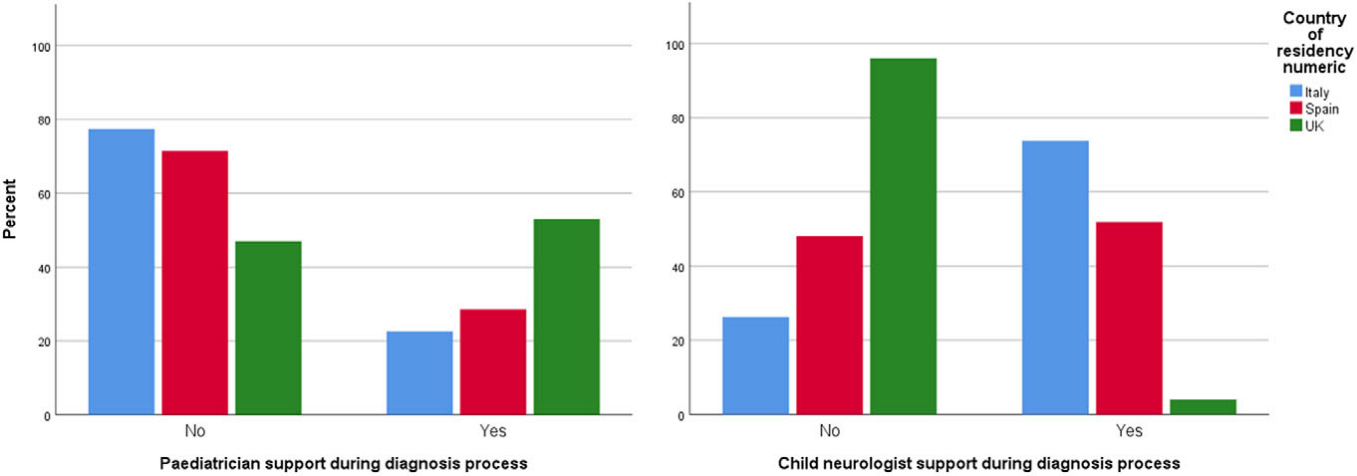
Percentages of autistic children who had a specialist assessment (pediatrician and child neurologists) as part of their autism diagnosis process, per country.

Of the 663 respondents, 626 completed the questions about professionals who assisted them during their children autism diagnosis process, of these 35% (23% in Italy, 29% in Spain, and 53% in the United Kingdom) stated that their children were seen by a pediatrician, whereas 41% (4% in the United Kingdom, 52% in Spain, and 74% in Italy) said that their children were seen by a child neurologist (Figure 1).

### Co-occurring diagnosis of epilepsy

Of the 663 respondents, 552 completed the questions about an associated epilepsy diagnosis. For specific country data, see Table 2.

**Table 2.** Number of respondents (and percentages) who stated that their autistic children had an associated diagnosis of epilepsy

Co-occurring diagnosis of epilepsy					
Country of residence		Italy	Spain	UK	Total
Yes	Number	7	30	18	55
	%	**6%**	**12%**	**10%**	**9%**
Total	Number	119	251	182	552

### Age of epilepsy diagnosis

The average age (in years) when epilepsy was diagnosed was 5.4 (SD = 3.7) in Spain, 7.17 (SD = 4.3) in Italy, and 10.44 (SD = 4.54) in the United Kingdom.

### Use of EEGs

**Figure 2.**
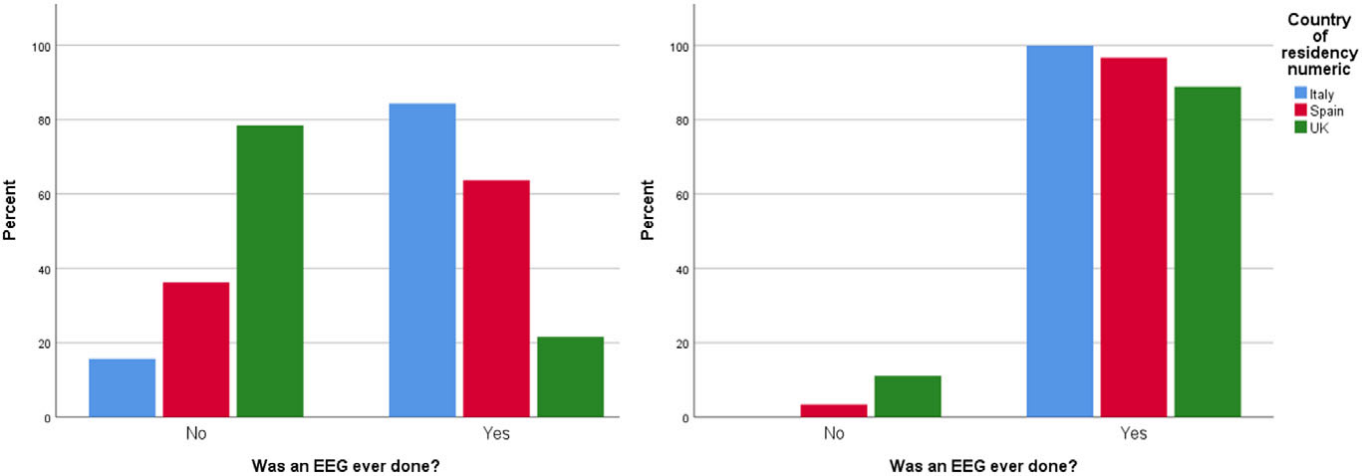
Percentages of electroencephalograms (EEGs) conducted in all children diagnosed with autism versus percentage of EEGs conducted in autistic children who were later diagnosed with associated epilepsy, per country.

Forty-four percent of the total survey respondents (79% in the United Kingdom, 36% in Spain, and 16% in Italy) said that their children were not screened for epilepsy with an EEG after autism diagnosis. However, the majority of respondents (95%) who said that their autistic children were later diagnosed with associated epilepsy (*n* = 55) stated that their children had an EEG done (Figure 2). The average age (in years) when these EEGs were done was 3.9 (SD = 1.95) in Italy, 4.5 (SD = 3.28) in Spain, and 9 (SD = 4.95) in the United Kingdom.

### Epilepsy treatment

Of the 55 caregivers who stated that their children had an associated diagnosis of epilepsy, 36 said that their autistic children were prescribed antiepileptic medication: 7 (100%) in Italy, 12 (67%) in the United Kingdom, and 17 (57%) in Spain. The average age (in years) when antiepileptic medication was initiated was 6.3 (SD = 3.98) in Spain, 6.5 (SD = 3.9) in Italy, and 10.4 (SD = 4.1) in the United Kingdom. 86% of respondents in Italy, 50% in Spain and 44% in the UK stated this medication was prescribed by a child neurologist.

Twenty-six of the 36 respondents provided us with specific information about the medication prescribed to their autistic children to treat epilepsy. All the respondents in Italy and the United Kingdom stated that their children were prescribed a single antiepileptic drug (monotherapy). In Spain, 57% of respondents stated that their children were prescribed a single antiepileptic drug, whereas 43% said that they were simultaneously prescribed multiple antiepileptic drugs.

### Co-ocurring diagnosis of intellectual disability (ID)

Of the 55 respondents who reported their children had an associated diagnosis of epilepsy, only 32 answered the questions about ID. For specific country data see Table 3.

**Table 3.** Number of respondents (and percentages) who stated that their autistic children who had an associated diagnosis of epilepsy also had a diagnosed intellectual disability (ID)

Other conditions diagnosed? Choice: ID					
Country of residence		Italy	Spain	UK	Total
Yes	Number	2	3	3	8
	%	29%	43%	17%	30%
Total	Number	7	7	18	32

## Discussion

Our investigation of the care pathway in autism and co-occurring epilepsy identified several barriers to an optimal care in Italy, Spain, and the United Kingdom. Among the most relevant identified gaps and their possible causes are the following:

1. *Absence of harmonized European clinical guidelines specific to the assessment and management of epilepsy associated with autism:*

Currently, the UK National Institute for Health and Care Excellence (NICE) provides evidence-based guidance for the diagnosis, support and management of autism in people aged under 19 years old, and separate evidence-based guidance for the diagnosis and management of epilepsy in Europe [14, 15, 23–25], but, to the best of our knowledge, there are no evidence-based harmonized clinical guidelines specific to the screening, diagnosis, and management of epilepsy associated with autism in Europe. This gap may be caused by the lack of awareness of the frequency of autism and epilepsy co-occurrence, which may also limit attention and allocation of resources by European health services to research in this area. We believe that the development of harmonized evidence-based clinical guidelines specific to the assessment and management of epilepsy associated with autism must be a clinical and research priority in Europe. We suggest autism, epilepsy experts (including Psychiatrists, Neuropaediatricians and neurologists) and members of the autistic community should be involved in the development of these guidelines.

1. *Inadequate screening for epilepsy in autistic children:*

NICE autism guidelines recommend considering coexisting disorders, such as epilepsy, during the autism diagnostic assessment process and if epilepsy is suspected to carry out appropriate assessments and referrals including an EEG. Scottish national clinical guideline number 145 for the assessment, diagnosis, and interventions for autism [15] states that medical investigations, including EEGs, should not routinely be performed but may be considered if there is suspicion of epilepsy. However, these guidelines do not clearly state which medical specialists should screen autistic children for epilepsy and decide if an EEG should be done. Nevertheless, the European Society for Child and Adolescent Psychiatry evidence-based recommendations for the diagnosis and management of autism [25] suggest that the autism diagnostic process should include a standard neurological examination conducted by a child neurologist if possible and that an EEG should be performed if epilepsy is suspected, but unfortunately these guidelines are not broadly followed in Europe as regional healthcare workers usually adhere to local protocols.

EEG abnormalities have been reported in autism since 1970 [26] with epileptiform activity with and without clinically obvious seizures reported in 20%–30% of autistic individuals [13, 27]. Additionally, researchers suspect that paroxysmal epileptiform discharges (EDs) without clinically apparent seizures may have neuropsychiatric and neurobehavioral consequences which can appear as cognitive, language, or behavioral changes and may worsen the clinical presentation of autism [28–30]. Furthermore, autistic children with evidence of EDs have an increased risk of developing epilepsy in adolescence [31].

In our study, only 41% of survey respondents stated that their children were assessed by a child neurologist, during the autism diagnostic process. Furthermore, 44% of survey respondents stated that their children’s healthcare providers did not screen the children for epilepsy using an EEG after autism diagnosis. These results highlight the lack of consensus regarding the medical specialists who should evaluate children for the presence of comorbidities, specifically epilepsy, during the autism diagnosis process and reflect the absence of clinical consensus about the need to conduct EEGs following an autism diagnosis and their appropriate timing and frequency. These gaps may be due to the lack of specific autism with associated epilepsy guidelines in Europe. For these reasons, we think that research on the cost-effectiveness of systematic epilepsy screening of all autistic children by an epilepsy specialist (e.g., pediatrician vs. child neurologist) who could then decide if an EEG is indicated, would be beneficial and would contribute to the development of autism with associated epilepsy guidelines.

1. *Lack of treatment of epilepsy in autistic children after diagnosis of epilepsy:*

NICE states that all people with a recent suspected seizure should be seen urgently by a specialist (i.e. a pediatrician with training and expertise in epilepsy) to ensure early treatment of epilepsy. We observed that 43% of autistic children with epilepsy in Spain and 33% in the United Kingdom were not prescribed any antiepileptic drugs, whereas in Italy 100% of them were on antiepileptic drugs. These discrepancies may be caused by the lack of harmonized guidelines which causes an inadequate screening and delayed diagnosis of epilepsy in autistic children. For example, emergency doctors in the UK may be referring autistic children with a suspected seizure to a paediatrician “with expertise in assessing first seizures and diagnosing epilepsy as recommended by NICE guidelines” but not formal neurology training while doctors in Italy or Spain maybe referring them to a child neurologist following ESCAP recommendations [14, 25]. There may also be clinical reasons explaining these discrepancies such as clinicians not prescribing medication in milder clinical cases while other clinicians prescribe medication after observing epileptiform activity in autistic children’s EEGs.This gap could as well be caused by insufficient availability of publicly funded paediatric epilepsy experts or child neurologists throughout the region.Additionally the lack of evidence on the effectiveness, cost-effectiveness and safety of anti-epilepsy treatment in autism [32] may be an issue here.

1. *Use of multiple antiepileptic drugs:*

NICE guidance on the diagnosis and management of epilepsies recommends that children, young people, and adults with epilepsy should be treated with a single antiepileptic drug (monotherapy) wherever possible; if the initial treatment is unsuccessful, monotherapy using another drug can then be tried [14]. Combination therapy (adjunctive or “add-on” therapy) should only be considered when attempts at monotherapy have not resulted in seizure freedom. If different combination therapies do not bring benefits, treatment should revert to the regimen (monotherapy or combination therapy) that has proved most acceptable in terms of providing the best balance between effectiveness in reducing seizure frequency and tolerability of side effects.

In our sample, 43% of caregiver respondents in Spain stated that their children were simultaneously prescribed multiple antiepileptic drugs, whereas 100% of respondents in Italy and the United Kingdom said that their autistic children were prescribed a single drug. This lack of consistency between countries may again follow from the lack of harmonized guidance and clinicians having to adhere to current epilepsy guidelines that do not cover the use of antiepileptic drugs in an autistic population [32].

Our findings need to be understood in the context of their limitations. First, calculating the rate of response for this survey it is not feasible as it is impossible to know the number of people who were reached by our posts in social media neither the number of people that received an invitation to complete the survey.Secondly, at the beginning or our epilepsy-specific questions, we did not clarify the difference between a seizure and epilepsy and only asked if the child had an associated diagnosis of epilepsy.Also, we did not ask for epilepsy specific questions such as severity, frequency of seizures or associated medical conditions which could affect the patient journey.Thirdly, we surveyed a convenience sample of people with internet access, active in social media and in contact with autistic associations; we do not know how well their responses represent the experience of all service users in Europe. Additionally, most respondents had a college degree or higher. Furthermore, the number of respondents who stated that their autistic children had a diagnosis of epilepsy and were on medication was small. Similarly, the number of respondents who answered our questions about co-morbidities, such as intellectual disabilities [6, 7, 8], was very small. Therefore, these results must be considered as a pilot study providing us with an initial, overall impression of the journey experienced by autistic children with associated epilepsy and their caregivers in Europe. Interpretation of survey data warrants follow-up or replication for confirmation in well-powered scientific studies that recruit larger samples. In future, researchers should also make extra efforts to recruit participants from diverse socioeconomic and educational backgrounds. Nevertheless, we hope that our findings highlight the current gaps in the care pathway of autism with associated epilepsy in Europe and help inform policy harmonization (Box 1) in order to improve the experiences and overall quality of life of autistic people with epilepsy and their caregivers.

Box 1. Policy recommendations.

- **Inform** health workers and parents/carers of autistic children about the frequent co-occurrence of autism and epilepsy.
- **Provide information** on seizures characteristics, crisis management, and basic life support to carers of autistic children.
- **Refer** the autistic child to an epilepsy specialist for **timely screening, diagnosis, and treatment of associated epilepsy.**
- If epilepsy is suspected, **perform an EEG.**
- **Develop harmonized evidence-based clinical guidelines specific** to the assessment and management of epilepsy associated with autism must be considered a clinical and research priority in Europe.
- **Avoid** the use of multiple antiepileptic drugs in autistic children; however, the choice of treatment should be made by the specialist (in agreement with carers) and based on seizure type and/or epilepsy syndrome.
- **Monitor** autistic children with epilepsy in a clinical setting at least twice a year.

## Supplementary material

For supplementary material accompanying this paper visit http://doi.org/10.1192/j.eurpsy.2023.2426. click here to view supplementary material
