# Supplementary material for: Autism with co-occurring epilepsy care pathway in Europe
Source: Eur Psychiatry. 2023 Jul 20;66(1):e61. doi: 10.1192/j.eurpsy.2023.2426 (PMC10486251; doi:10.1192/j.eurpsy.2023.2426)
Supplement: Supplementary file 1 [file S0924933823024264sup001.docx]

**Supplementary material.**

**Appendix 1: Summarized survey in English (including sample characterization questions and epilepsy specific questions).**

**BACKGROUND INFORMATION:**

**These questions should be answered by the parent/carer of the person on the autism spectrum.**

The questions in this initial section will ask you about your sex, where you live, your age, your education, the current monthly income of the household where the child with autism lives and the number of people living in the home.

1. Please state your country of residence
2. County
3. Town/City
4. Is one of the parents a migrant? Yes/No. Country of origin:
5. Your biological sex (sex assigned at birth)

Male ⭘

Female ⭘

1. How old are you?
2. Who is answering this questionnaire?

I am a parent of a person on the autism spectrum ⭘

I am a person on the autism spectrum and a parent of a person on the autism spectrum ⭘

I am a carer of a person on the autism spectrum (not a blood relative) ⭘

I am a blood relative of a person on the autism spectrum (not a parent).

Sibling ⭘

Other ⭘

Please specify:

1. Approximately how many people live in your city/town?

Fewer than 10,000 ⭘

Between 10,000 and 50,000 ⭘

Between 50,000 and 150,000 ⭘

Between 150,000 and 1,000,000 ⭘

More than 1,000,000 ⭘

1. Please indicate the highest level of education completed in your household (by you or your partner).

No formal education completed ⭘

Primary education (or similar: elementary, middle school, etc.) ⭘

Secondary School (high school) ⭘

Further education (College) ⭘

Undergraduate degree (Bachelor’s) ⭘

Postgraduate Education (Master’s, PhD, etc.) ⭘

1. How many people live in the same household as the child with autism?
2. Optional question: Please state your approximate current monthly household income (in your country’s currency).

< 500 € ⭘

Between 500-1000€ ⭘

1000-1500€ ⭘

1500-2000€ ⭘

>2000€ ⭘

Without income ⭘

I would rather no answer ⭘

1. What is the age of the child with autism?
2. What is the biological sex (assigned at birth) of the child with autism?

Male ⭘

Female ⭘

1. What is the verbal ability of the child with autism?

Does not talk ⭘

Uses single words only (e.g. “daddy”, “mommy”) ⭘

Uses two or three word phrases (e.g. “want biscuit”) ⭘

Uses sentences with four or more words (e.g. “I want a biscuit”) ⭘

Uses complex sentences (e.g. “When we get home, can I have a biscuit?”) ⭘

**EPILEPSY-SPECIFIC QUESTIONS:**

These questions need to be answered only if the child or you have experienced seizures/fits or have been diagnosed with epilepsy.

1. Has the child been diagnosed with epilepsy?

Yes ⭘

No ⭘

<If your answer is No please skip to question 67>

1. At what age was the child diagnosed with epilepsy?
2. Is the child on medication to treat epilepsy?
3. At what age was the child prescribed medication for the treatment of epilepsy?
4. Who prescribed this medication?

Psychiatrist ⭘

Child neurologist ⭘

Neurologist ⭘

Other ⭘

Please specify: _____________

1. If this was privately funded, how much does each appointment cost you?
2. How often do you need to see this doctor?
3. If on medication, please complete this table:

| Name of drug | Dosage (if known) | Dose frequency | Paid for directly by carer (Yes/No) | If yes, how much does it cost per month? (£) |
| --- | --- | --- | --- | --- |
|  |  |  |  |  |
|  |  |  |  |  |
|  |  |  |  |  |

**FINAL QUESTION**

1. Is there something else you want to tell us that we didn’t cover during this survey?

|  |
| --- |

End of questionnaire

Thank you for your participation!
